# Deep Learning outperforms physicians in myopathy and neuropathy classification based on Needle Electromyography Signal

**DOI:** 10.1371/journal.pone.0339691

**Published:** 2026-05-19

**Authors:** Ilhan Yoo, Jaesung Yoo, Dongmin Kim, Ina Youn, Hyodong Kim, Michelle Youn, Jun Hee Won, Woosup Cho, Youho Myong, Sehoon Kim, Ri Yu, Sung-Min Kim, Kwangsoo Kim, Seung-Bo Lee, Keewon Kim

**Affiliations:** 1 Department of Neurology, Nowon Eulji Medical Center, Eulji University School of Medicine, Nowon-gu, Seoul, Republic of Korea; 2 School of Electrical Engineering, Korea University, Seongbuk-gu, Seoul, Republic of Korea; 3 Biomedical Research Institute, Seoul National University Hospital, Jongno-gu, Seoul, Republic of Korea; 4 Department of Computer Science, New York University, New York, New York, United States of America; 5 Department of Rehabilitation Medicine, Seoul National University Hospital,‌‌ Jongno-gu, Seoul, Republic of Korea; 6 Department of Neurology, Seoul National University Hospital, Seoul National University College of Medicine, Jongno-gu,‌‌ Seoul, Republic of Korea; 7 Transdisciplinary Department of Medicine & Advanced Technology, Seoul National University Hospital, Jongno-gu, Seoul, Republic of Korea; 8 Department of Medical Informatics, Keimyung University School of Medicine, Nam-gu, Daegu, Republic of Korea; 9 Office of Vision, Seoul National University College of Medicine, Jongno-gu, Seoul, Republic of Korea; Fondazione Policlinico Universitario Gemelli IRCCS, ITALY

## Abstract

Needle electromyography (nEMG) is a valuable tool for diagnosing patients with neuromuscular diseases. However, it is labor-intensive and is prone to diagnostic inaccuracies stemming from human biases. To address these challenges, we validated an nEMG diagnosis-aiding system with minimal preprocessing using deep learning model to classify patients into three categories: normal, myopathy, and neuropathy. Using 376 nEMG signals from 57 patients from a tertiary university hospital database through nested k-fold cross validation, deep learning model surpassed the classification performance of six electromyographers. The median patient classification accuracy, precision, sensitivity, and specificity of the deep learning model was 0.70, 0.70, 0.70, and 0.85, respectively, whereas those of the physicians were 0.55, 0.60, 0.54, and 0.78, respectively. Model interpretability and failure analysis showed that the deep learning model classifies based on relevant signal features. Despite higher accuracy of DL model, the number of unanimously misclassified cases were higher in the DL model than physicians. Our study validates deep learning is a fast, accurate, and practical application to aid physicians in diagnosing patients using nEMG signals.

## Introduction

Needle electromyography (nEMG) is an essential electrophysiological measurement that is utilized as a diagnostic test, to diagnose neuromuscular diseases. It is used alongside clinical evaluation, serum studies, tissue biopsy, and genetic testing, with converging evidence supporting the diagnosis. In nEMG, a needle electrode is inserted into a muscle and records motor unit action potentials (MUAP) generated by the nerves, muscles, and neuromuscular junctions during volitional state [1–6]. The nEMG signal, comprised of MUAPs, reflects the anatomical and physiological states of peripheral nerves and muscles, and its signal abnormalities are used to diagnose neuropathy and myopathy [1–6]. Patients with neuropathy commonly exhibit MUAPs with large amplitudes, long durations, and reduced recruitment, whereas those with myopathy commonly exhibit MUAPs with small amplitudes, short durations, and early recruitment [1,5–12]. In clinical settings, MUAP could plays a critical role in distinguishing neuropathy and myopathy as their symptoms are sometimes similar [1,5–12].

Despite the important role of nEMG in distinguishing neuromuscular diseases, nEMG evaluation possesses some limitations. First, the accuracy of nEMG evaluation is reliant on the examiner’s proficiency in which the reliability may vary across examiners by 62–81% [13]. Second, the manual evaluation of nEMG signal abnormalities is labor intensive. The rising incidence of neuromuscular diseases increases pressure on physicians as more patients require nEMG evaluations [14–17]. Thus, developing a precise and efficient method for interpreting nEMG data could aid rapid and accurate diagnoses by physicians.

In recent years, deep learning (DL) [18–21] has been used to develop fast and efficient prediction models by leveraging large clinical datasets including electrocardiographic and electroencephalographic data [22–25]. Studies have shown that the clinical performance of the DL model was comparable to or surpassed that of humans [26–28]. While there have been DL studies with EMG data [29–34], the performance of DL in analyzing MUAP signals and validating its clinical practicality remains unknown.

To lift the practical challenges of nEMG diagnosis, we validated an nEMG diagnosis-aiding system with a DL model that classifies patients into myopathy, neuropathy, or normal state based on nEMG signals during volitional state. After training our DL model, we also investigated how our DL model is making decisions with an interpretability tool.

## Materials and methods

### Study design and preparation

We retrospectively reviewed the electronic medical records of individuals who visited Seoul National University Hospital and underwent nEMG between June 10th 2015 and August 10th 2020. The queried data contained identifiable patient IDs, which were excluded from all downstream analyses. Patients who showed chronic symptoms for at least three months were selected. All myopathic patients underwent biopsy, and those with congenital myopathy also received genetic testing. Polyneuropathy in neuropathic patients was diagnosed based on clinical symptoms, nerve conduction studies, and MRI scans. This study was approved by the Institutional Review Board (IRB) of Seoul National University Hospital (No. 2008-055-1147) and informed consent was not obtained because the study was retrospective. The data was accessed throughout the study period, from February 1st 2021 to August 31st, 2024.

nEMG measurement was performed using the Nicolet EDX EMG system and monopolar needle electrode (Natus, Middleton, WI, USA). The filter was set at 20 Hz (low cut) and 10 kHz (high cut). The nEMG signals were recorded with a sampling rate of 48 kHz. Target muscle was selected by certified neurologists or physiatrists. The nEMG electrophysiological diagnosis is as follows. The signal during resting state was inspected for several seconds after initially inserting a needle. Subsequently, signal during minimal, moderate, and maximal muscle contractions was inspected. The diagnosis of the patient was determined comprehensively considering duration and amplitude of individual MUAP, and the recruitment and interference pattern of MUAPs. The nEMG signal during muscle contraction was saved to the system and the resting potentials were discarded. Signal artifacts from needle insertion or patient movements at the beginning and the end of the signal were removed.

**Fig 1:**
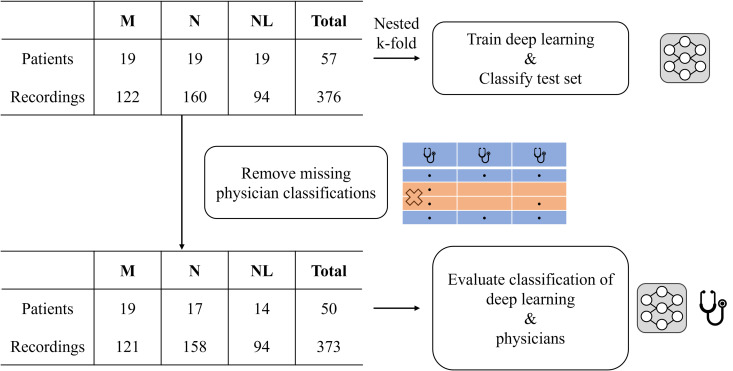
Summary of needle electromyography dataset. The whole dataset was divided in nested k-fold split to train the deep learning model and classify on the test set. The patients and muscle signals without physician classification labels were discarded in evaluating physician and deep learning performance. Abbreviations: M (Myopathy), N (Neuropathy), NL (Normal).

Initially, 20 patients each diagnosed with myopathy, neuropathy, and normal were randomly selected from the database, resulting with 60 patients in total. After manual curation by certified neurologists and physiatrists, one myopathic and neuropathic patient were excluded due to atypical disease characteristics and poor signal quality. Additionally, one normal-class patient was removed because all signal measurements were shorter than the required 0.4 seconds minimum window for the DL model classification. Certified neurologists and physiatrists reviewed the retrospective nEMG and patient information data, confirming the diagnoses for all patients to ensure reliable DL training as well as DL and physician evaluation. In total, there were 19 patients each diagnosed with myopathy, neuropathy, and normal, total of 57 patients. (Fig 1). The 57 patients were used in DL training and inference.

During evaluation of the DL model and the physicians, the missing classification labels of physicians for signals and patients were removed. The diagnosis classification task is divided into signal classification and patient classification, where accurate patient classification is the goal. The diagnosis labels of each signal follow that of the patient. Patients and signals with any missing classification labels from the physician’s assessment were removed from evaluation. Total of 50 patients and 373 signals were used to evaluate the performance of DL model and the physicians. The summary of the dataset is shown in Fig 1 and the demographic characteristics are presented in Table 1.

**Table 1:** Demographic characteristics of the patients. Abbreviations: SD (Standard deviation), NA (Not applicable).

Feature	Patient diagnosis			p-value
	Myopathy	Neuropathy	Normal	
N	19	19	19	NA
Female, n (%)	14 (73.7)	12 (63.2)	12 (63.2)	0.729
Age in years, median [Q1, Q3]	54.0 [35.0, 66.5]	60.0 [51.5, 68.5]	62.0 [51.0, 72.0]	0.383
Diagnosis (n)	Idiopathic generalized myopathy (11)	Diabetic polyneuropathy (1)	NA	NA
	Statin-induced toxic myopzathy (1)	Polyradiculopathy (1)		
	Limb girdle muscular dystrophy (1)	Median and ulnar neuropathy around the wrist (1)		
	Inflammatory myopathy (2)	Brachial plexopathy (7)		
	Emery–Dreifuss muscular dystrophy or dysferlinopathy (1)	Lumbosacral radiculopathy (3)		
	X-linked FHL1-related myopathy (1)	Cervical radiculopathy (2)		
	Steroid-induced toxic myopathy (1)	Critical illness polyneuropathy (1)		
	Critical illness myopathy (1)	Chemotherapy-induced polyneuropathy (1)		
		Postpolio syndrome (1)		
		Progressive muscular atrophy (1)		
Number of signals	122	160	94	
Number of signals per patient, median [Q1, Q3]	6.0 [5.0, 7.0]	7.0 [5.5, 10.5]	5.0 [3.5, 5.5]	0.012
Total signal length, s	312.8	422.8	203.5	
Total signal length per patient, median [Q1, Q3]	14.4 [11.8, 16.7]	18.6 [12.6, 30.5]	11.2 [8.7, 11.8]	0.001
Mean signal length in seconds, median [Q1, Q3]	2.6 [1.8, 3.4]	2.9 [1.9, 3.4]	2.2 [1.2, 2.9]	<0.001
Mean signal length per patient, median [Q1, Q3]	2.7 [2.1, 2.9]	2.8 [2.6, 2.8]	2.3 [2.0, 2.7]	0.237

### Classification by the deep learning model

The nEMGNet and divide-and-vote (DiVote) algorithm was used to classify the diagnosis of a patient [34]. The nEMGNet is a fast DL model optimized for diagnosis classification based on raw nEMG signals with minimal preprocessing, and the DiVote algorithm allows to classify the diagnosis of a patient given heterogeneous types, numbers, and duration of muscle signals per patient. The method have shown stable performance and explainability [34] with the highest score of The Checklist for Artificial Intelligence in Medical Imaging (CLAIM) among existing nEMG classification methods, which is a guideline for developing artificial intelligence for medicine [35,36].

The following hyperparameters were used for the nEMGNet and DiVote classifier. The nEMG data were down-sampled to 10 kHz for computational brevity. The nEMGNet-B with 2 residual blocks was used as the DL model. Early stopping [37] was performed by evaluating the accuracy of the validation set every 30 updates and the patience value was set to 100. Cross-entropy loss [38] was used as the loss function with weights inversely proportional to the number of training segments per class. Learning rate of 1e-3, and a batch size of 32 was used in training. Performance was measured through 5 × 3-fold nested cross validation where 5 outer folds were used as test set and the 3 inner folds were used as train and validation set. The nested k-fold cross validation validates the DL model more rigorously compared to conventional k-fold cross validation. Total of 3 DL model with different random seeds were trained for each fold combination, resulting in 9 DL classification results for test set from 3 random seeds and 3 validation folds.

After training and performance evaluation, the characteristic signal patterns that the DL model has learned for each disease type were inspected to ensure the model is classifying disease types based on relevant MUAP patterns. The feature visualization [39] was used which is an explainable DL method.

### Classification by physicians

A web-based nEMG signal labelling platform was developed for classification by physicians. Two neurologists and four physiatrists with more than five years and 1000 cases of nEMG interpretation experience were recruited to annotate each nEMG signal and patient without additional clinical information (6 physicians in total). The physicians were informed about which nEMG muscle signals corresponded to each anonymized patient, allowing them to classify the individual muscle nEMG signals and patients accordingly.

### Evaluation

The signal and patient classification performance of the DL model and physicians were measured with the following metrics: accuracy, positive predictive value (precision), sensitivity (recall), specificity, and F1 score. All metrics except accuracy are binary classification metrics which were measured by using the one-versus-rest method for each class and averaged over all classes. The area under the receiver operating characteristic curve (AUROC) was not measured during one-vs-rest binary classification evaluation, as threshold-based binary classification performance was not the focus, which is the primary purpose of AUROC. The metrics were calculated using the following formulas:


$$\begin{array}{c} Accuracy=\frac{\left(TP+TN\right)}{\left(TP+TN+FP+FN\right)} \end{array}$$ (1)

$$\begin{array}{c} Precision=\frac{TP}{TP+FP} \end{array}$$ (2)

$$\begin{array}{c} Recall=\frac{TP}{TP+FN} \end{array}$$ (3)

$$\begin{array}{c} Specificity=\frac{TN}{TN+FP} \end{array}$$ (4)

$$\begin{array}{c} F1=\frac{2\times Precision\times Recall}{Precision+Recall} \end{array}$$ (5)

Failure analysis of the DL model and physicians were performed by inspecting signals with highly consistent signal classification labels among DL models and physicians. The signals with 8 out of 9 DL models or 5 out of 6 physicians classifying into the same label were considered as highly consistent. The signals predicted as correct class by the DL model and incorrect class by the physicians were inspected, and vice versa.

Statistical analyses were performed using R statistical software (version 4.1.0; R Foundation for Statistical Computing, Vienna, Austria) and Python programming language (version 3.6; Python Software Foundation, Delaware, United States) with the tableone library [40]. Normal distribution for the continuous variables was assessed using the Shapiro–Wilk test. Differences in categorical and continuous variables across myopathy, neuropathy, and normal states were assessed using Pearson’s chi-square and Kruskal–Wallis tests, respectively. Statistics are expressed as the mean±standard deviation for continuous variables and as a number (%) for categorical variables. A p-value < 0.05 was considered statistically significant.

## Results

### Signal classification

The signal classification scores of the DL model and physicians are shown in Table 2. The median accuracy of the DL model (0.61) was higher than that of physicians (0.54) with statistical significance (p = 0.045). The ROC curve shows the DL model and physicians’ per-class classification results are similar for myopathy and neuropathy, while the DL model performs better for normal signals (S1 Fig). The precision-recall curve shows the physicians outperform the DL model for myopathy signals whereas the DL model outperforms physicians for normal signals.

**Table 2:** Signal classification scores of the deep learning model and physicians. Deep learning model scores are measured with 9 scores from 3 validation folds and 3 random seeds. Physicians scores are measured with 6 scores from 6 physicians. The full score is listed in S1 File.

	Deep learning model (median [Q1, Q3])	Physicians, (median [Q1, Q3])	p-value
Accuracy	0.61 [0.61, 0.62]	0.54 [0.47, 0.59]	0.045
Precision (Positive predictive value)	0.61 [0.59, 0.61]	0.59 [0.57, 0.63]	0.906
Recall (Sensitivity)	0.60 [0.59, 0.60]	0.51 [0.46, 0.56]	0.045
Specificity	0.80 [0.80, 0.81]	0.76 [0.73, 0.79]	0.059
F1-score	0.60 [0.59, 0.61]	0.50 [0.47, 0.56]	0.045

The per-class accuracy of myopathy, neuropathy, and normal signals by the DL model were 68.04±4.99%, 63.85±4.18%, and 47.28±8.69% respectively, and 40.91±12.23%, 68.99±9.86%, and 46.45±15.64% by the physicians, respectively (S2 Fig). The DL model classified myopathy, neuropathy, and normal signals with descending per-class accuracy, while the order for physicians were neuropathy, normal, and myopathy. The normal label signals misclassified as neuropathy by physicians were notably high with 50.71±16.02%, which accounts for the physicians’ bias to classify signals as neuropathy. The full signal classification results and scores are can be found in S1 File and S3 File, respectively.

### Patient classification

**Fig 2:**
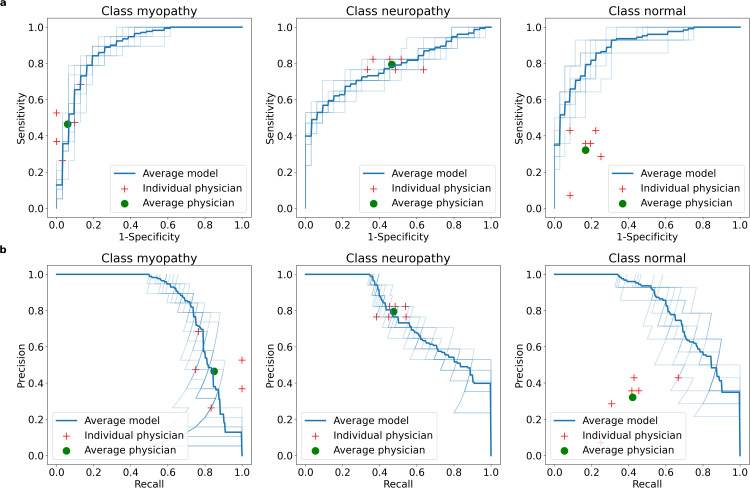
Per-class receiver operating characteristic and precision-recall curves of the physicians and the deep learning model in patient classification. **(a)** Receiver-operating characteristic curve. **(b)** Precision-recall curve.

The patient classification scores of the DL model and physicians are shown in Table 3. The median accuracy of the DL model (0.70) was higher than that of physicians (0.55) with statistical significance (p = 0.001). The ROC curve and precision-recall curve shows that the DL model and physicians’ per-class classification results are similar for myopathy and neuropathy, while the DL model performs better for normal label patients (Fig 2).

**Table 3:** Patient classification scores of the deep learning model and physicians. Deep learning model scores are measured with 9 scores from 3 validation folds and 3 random seeds. Physicians scores are measured with 6 scores from 6 physicians. The full score is listed in S2 File.

	Deep learning model (median [Q1, Q3])	Physicians, (median [Q1, Q3])	p-value
Accuracy	0.70 [0.70, 0.72]	0.55 [0.48, 0.56]	0.001
Precision (Positive predictive value)	0.70 [0.69, 0.73]	0.60 [0.54, 0.64]	0.002
Recall (Sensitivity)	0.70 [0.69, 0.72]	0.54 [0.48, 0.55]	0.001
Specificity	0.85 [0.84, 0.86]	0.78 [0.74, 0.78]	0.001
F1-score	0.70 [0.69, 0.72]	0.53 [0.45, 0.55]	0.001

**Fig 3:**
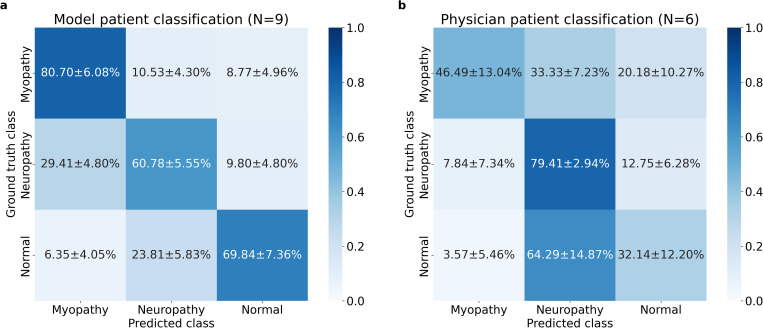
Confusion matrices of the deep learning model and physicians in patient classification. Entries indicate mean ± standard deviation. **(a)** Classification by deep learning models. **(b)** Classification by physicians.

The per-class accuracy of myopathy, neuropathy, and normal patients by the DL model were 80.70±6.08%, 60.78±5.55%, and 69.84±7.36% respectively, and 46.49±13.04%, 79.41±2.94%, and 32.14±12.20% by the physicians, respectively (Fig 3). The DL model classified myopathy, normal, and neuropathy label patients with descending per-class accuracy, while the order for physicians were neuropathy, myopathy, and normal. The normal label patients misclassified as neuropathy by physicians were notably high with 64.29±14.87%, which accounts for the physicians’ bias to classify patients as neuropathy as well as the signals. The full patient classification results and scores are can be found in S2 File and S4 File, respectively.

The median accuracy of the DL model is significantly higher (p < 0.001) for patient classification (0.70) than signal classification (0.61) which is an observed improvement from previous work [34]. However, the median accuracy of physicians were not significantly higher (p = 1.00) for patient (0.55) and signal (0.54) classification.

### Learned features of the deep learning model

**Fig 4:**
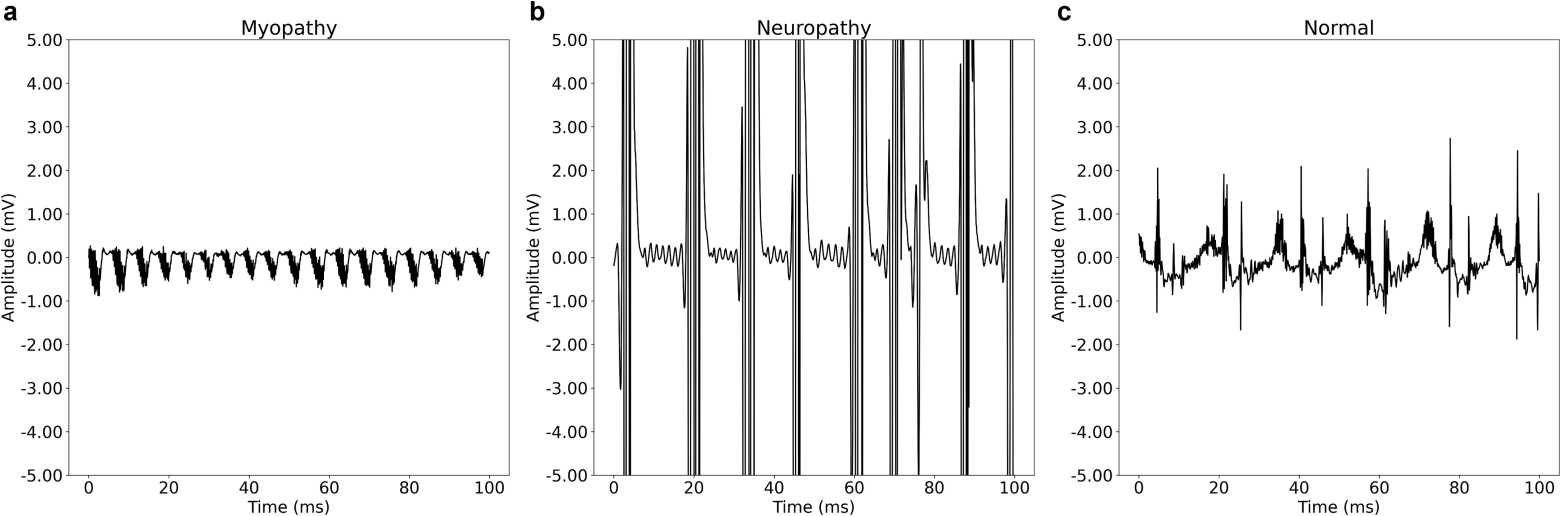
Example signals that the deep learning model is most likely to predict as belonging to each class. The signals are generated artificially using feature visualization.

The signals generated from feature visualization represent the signals that the DL model perceives most likely as myopathy, neuropathy, or normal (Fig 4). The signal characteristics the DL model has learned to capture were visually similar to the typical characteristic nEMG signals of neuropathy, myopathy, and normal states. Waveforms that were interpreted as myopathy to the DL model showed early recruitment patterns (small amplitudes and short durations, Fig 4a), whereas those interpreted as neuropathy showed delayed recruitment patterns (high amplitudes and long durations, Fig 4b). Thus, the feature visualization results validated that the DL model predictions were based on relevant features not artifacts.

### Failure analysis of the deep learning model and physicians

**Fig 5:**
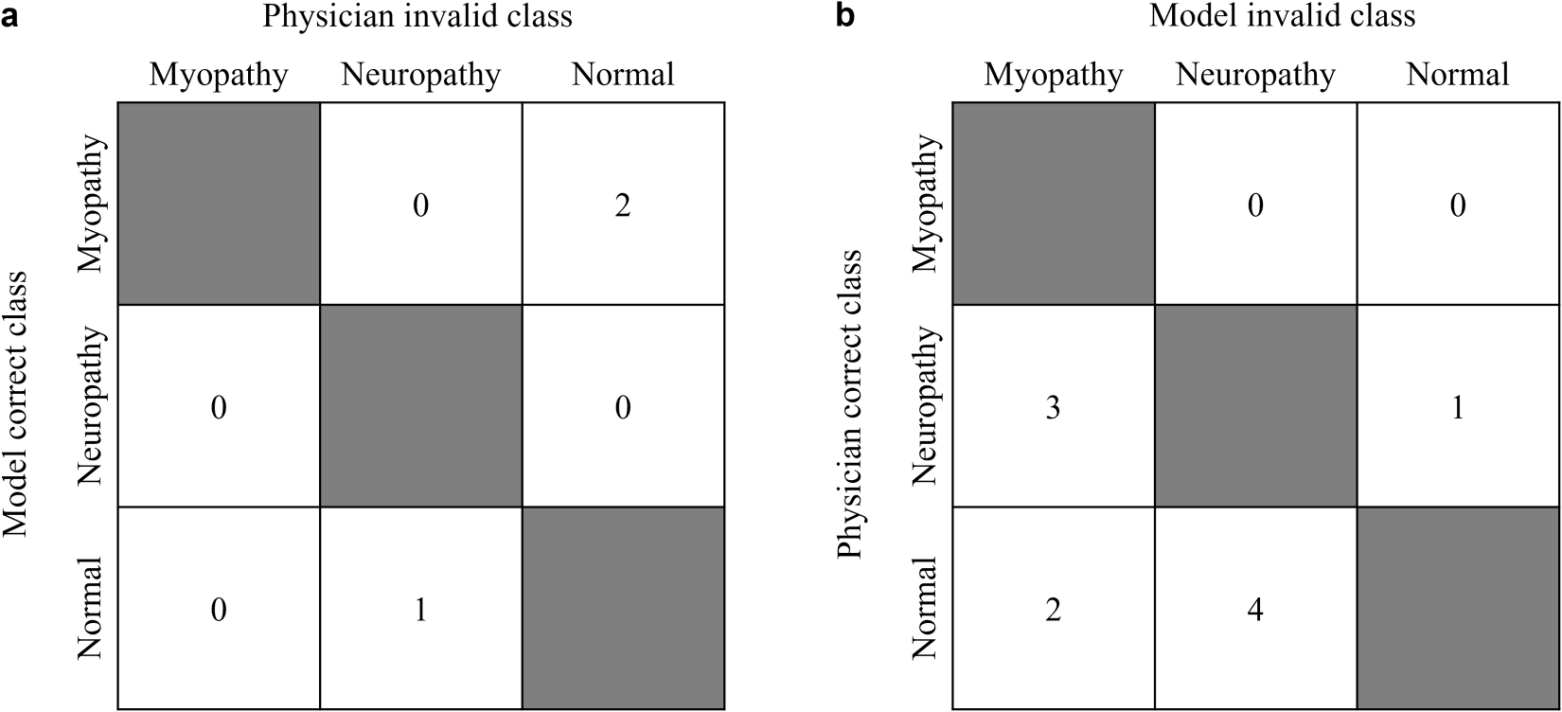
Number of misclassified signals. The entries indicate the number of signals. **(a)** Signals that the majority of deep learning model classified correctly and majority of physicians misclassified. **(b)** Signals that majority of physicians classified correctly and majority of deep learning model misclassified. Majority is defined as 8 out of 9 deep learning models or 5 out of 6 physicians.

To inspect the vulnerability of the DL model and the physicians in recognizing the signal patterns, the misclassified signals were investigated where majority of DL model classified correctly and physicians did not, and vice versa (Fig 5). While the signal classification accuracy of the DL model was higher than the physicians (Table 2), the majority of DL models misclassified 10 signals which more than how majority of physicians misclassified 3 signals. This can be explained by physicians having inter-rater variability [13] while the DL models tend to be unanimous in their prediction results.

**Fig 6:**
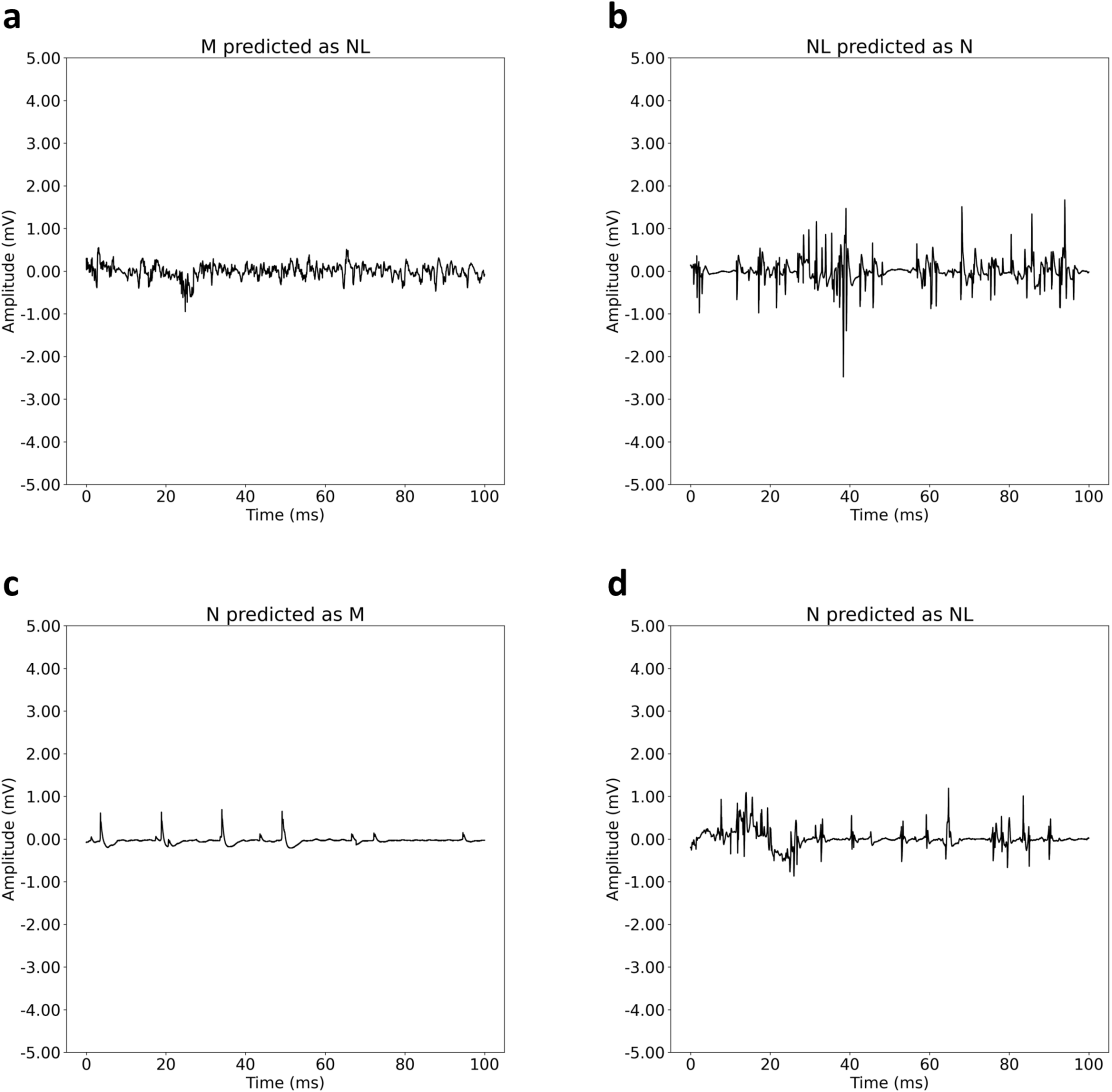
Misclassified signals. **(a)-(b)**: Signals that majority of deep learning model classified correctly and majority of physicians misclassified. **(c)-(d)**: Signals that majority of physicians classified correctly and majority of deep learning model misclassified. Abbreviations: M (Myopathy), N (Neuropathy), NL (Normal).

Among the signals which majority of the DL model were correct but the physicians were not (Fig 5a), the myopathic signal misclassified as normal shows many motor units with small amplitude and early recruitment which indicates myopathic characteristics (Fig 6a). The normal signal misclassified as neuropathy might be interpretted as neuropathy from its large amplitude and the featues that look like neuropathic MUAP caused by needle insertion noise, but the recruitment and inference patterns indicate normal characteristics which the DL model captured when the physicians could not (Fig 6b).

Among the signals which majority of the physicians were correct but the DL model were not (Fig 5b), the neuropathic signal misclassified as myopathy shows reduced recruitment, a characteristic of neuropathy, along with small amplitude (Fig 6c). The physicians considered the recruitment patterns and amplitude while the DL model may have focused on the small amplitude. This signal appears to be measured in a poor volitional state, indicative of a patient with severe neuropathy, which contrasts with other signals in the DL training data that were recorded with adequate volition. The neuropathic signal misclassified as normal may have some characteristics of normal signal caused by needle insertion noise, but the large amplitude and reduced ‌‌recruitment indicates neuropathy characteristics (Fig 6d).

## Discussion

The results of our study indicate that the DL model can classify myopathy, neuropathy, and normal patients with higher performance than physicians, based on nEMG signals during volitional state. The algorithm mitigates the heterogeneity of patients’ signal types and quantities [34], an important step in classifying neuromuscular disease of patients [36]. The algorithm uses raw data with no preprocessing, which makes it practical and applicable to clinical assistance. The DL model classifies signals in under 1 second [34], supporting clinical applicability and potential real-time predictions. Additionally, the algorithm’s high CLAIM score underscores its rigor among recent nEMG disease classification algorithms [36]. The post-hoc analysis with feature visualization indicates that the DL model is capturing signal characteristics that resemble each disease type (Fig 4), and the misclassified signals by the DL model indicate relatively ambiguous features that the model could improve upon capturing (Fig 6). Therefore, our findings suggest that the DL model is an accurate, fast, and relatively stable method for aiding EMG diagnosis.

The classification pattern of physicians was certainly different from the DL model. First, the patient classification accuracy of 0.55 was lower than expected. We find the reason from the study cohort where 19 patients were from each myopathy, neuropathy, and normal class. The disease type prevalence of the cohort was considerably higher than the real-world prevalence of approximately 200 per 100,000 individuals [41]. Additionally, the distribution of patients diagnosed with myopathy, neuropathy, and normal at Seoul National University Hospital was 61 (0.8%), 3544 (47.9%), and 3791 (51.3%), respectfully. This ratio differs from that of our study cohort even within a hospital setting. Physicians may be less familiar with classifying less prevalent neuromuscular diseases, such as myopathy. This study highlights this bias, which can be addressed to enhance diagnostic accuracy. Second, the classification by physicians differed from the real-world diagnostic process. Physicians usually consider both the nEMG signals and additional patient information holistically, such as patient history, demographics, and symptoms, which were absent in this study. Third, there was no significant performance improvement from signal classification to patient classification (Table 2,3)). The physicians classified the patients based on signal classification, potentially biasing them to assign the same class to both signals and patients. In contrast, the algorithm classified the patient by integrating the signal classification results independently. Finally, the physicians had a lower consensus in their predictions than the DL model resulting in smaller number of misclassified signals by majority of the raters (Fig 5). This indicates that the inter-rater variability actually works in favor for reducing misclassification among many raters, working like a buffer when new or ambiguous signal is presented, which is a caveat DL model presents from its high consensus prediction results.

The study has several limitations, suggesting important future directions for research. First, an external validation could provide a stronger evidence for the effectiveness of the nEMG diagnosis-aiding system. Evaluating both physicians and the DL model on highly curated data is more critical than relying solely on large datasets, as performance assessment becomes difficult if the test data is unreliable. While our data is highly curated and provides a robust foundation for training a DL model and evaluating both DL model and physicians, including data from multiple institutions or using prospective data could enhance the model’s applicability as a diagnostic aid for neuromuscular diseases.

Second, a larger nEMG dataset could help validate the stability and performance of the DL model. We employed nested k-fold cross validation, a more rigorous approach than conventional k-fold cross-validation, to address the small sample size and ensure the model’s generalizability. Nevertheless, a larger curated nEMG cohort could further confirm the DL model’s performance. Additionally, larger cohort would likely include more diverse subtypes of neuromuscular diseases such as inclusion body myositis, which shows both neuropathic and myopathic features simultaneously in nEMG. This would enable the development of a classifier model with detailed disease subtypes [42].

In conclusion, we demonstrated that a simple and fast DL method can analyze nEMG signals with higher signal and patient classification accuracy than physicians. Although the DL model misclassifies based on ambiguous and relevant signal features, it also exhibited more unanimously misclassified signals than physicians. Therefore, under physician supervision to ensure safety, the DL model can serve as an effective tool to accelerate diagnosis [43].

## Supporting information

S1 Fig Per-class receiver operating characteristic and precision-recall curves of the physicians and the deep learning model in signal classification. (a) Receiver-operating characteristic curve. (b) Precision-recall curve.
(PNG)

S2 Fig Confusion matrices of the deep learning model and physicians in signal classification. Entries indicate mean ± standard deviation.
(PNG)

S1 File Full signal classification scores. (XLSX)

S2 File Full patient classification scores. (XLSX)

S3 File Signal classification results. (XLSX)

S4 File Patient classification results. (XLSX)
