# Supplementary figures and images for: Deep Learning outperforms physicians in myopathy and neuropathy classification based on Needle Electromyography Signal
Source: PLoS One. 2026 May 19;21(5):e0339691. doi: 10.1371/journal.pone.0339691 (PMC13186374; doi:10.1371/journal.pone.0339691)

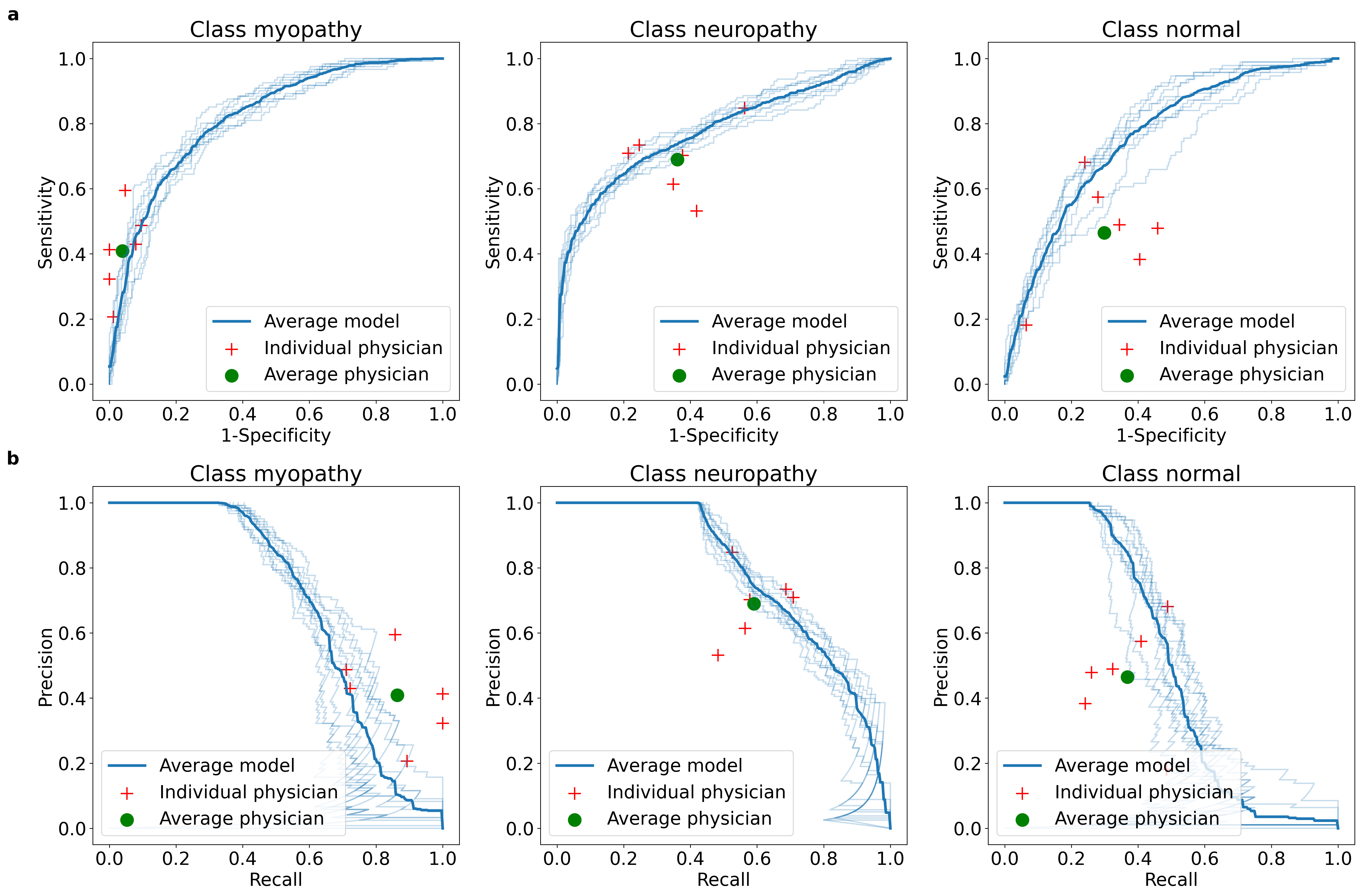

Supplement: S1 Fig — (a) Receiver-operating characteristic curve. (b) Precision-recall curve. (PNG) [file pone.0339691.s001.png]

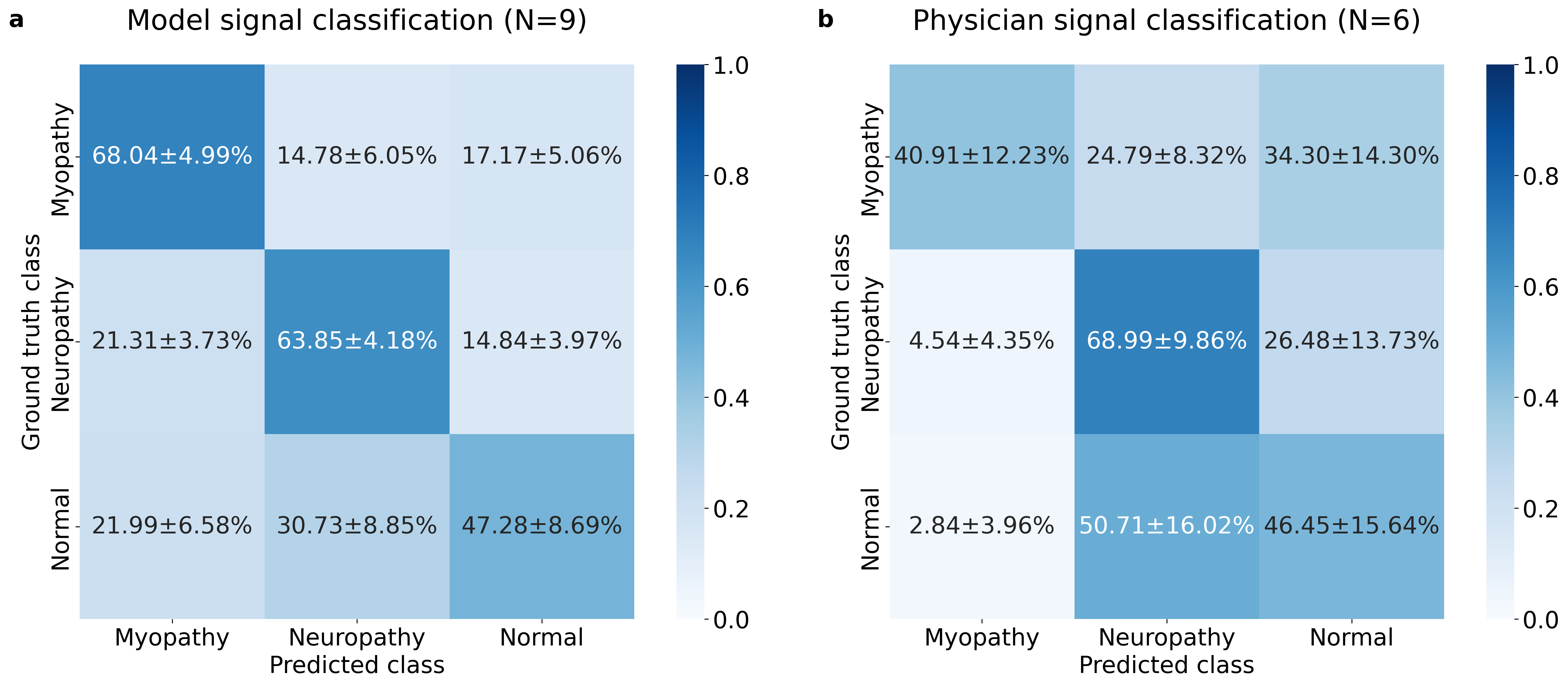

Supplement: S2 Fig — Entries indicate mean ± standard deviation. (PNG) [file pone.0339691.s002.png]
